# Development of a New Hyaluronic Acid Based Redox-Responsive Nanohydrogel for the Encapsulation of Oncolytic Viruses for Cancer Immunotherapy

**DOI:** 10.3390/nano11010144

**Published:** 2021-01-08

**Authors:** Siyuan Deng, Alessandra Iscaro, Giorgia Zambito, Yimin Mijiti, Marco Minicucci, Magnus Essand, Clemens Lowik, Munitta Muthana, Roberta Censi, Laura Mezzanotte, Piera Di Martino

**Affiliations:** 1 School of Pharmacy, University of Camerino, Via S. Agostino 1, 62032 Camerino, Italy; siyuan.deng@unicam.it (S.D.); roberta.censi@unicam.it (R.C.); 2 Medical School, University of Sheffield, Beech Hill Road, Sheffield S10 2RX, UK; ale10iscaro@gmail.com (A.I.); m.muthana@sheffield.ac.uk (M.M.); 3 Department of Radiology and Nuclear Medicine, Erasmus Medical Center, 3015 GD Rotterdam, The Netherlands; g.zambito@erasmusmc.nl (G.Z.); c.lowik@erasmusmc.nl (C.L.); l.mezzanotte@erasmusmc.nl (L.M.); 4 Department of Molecular Genetics, Erasmus Medical Center, 3015 GD Rotterdam, The Netherlands; 5 Physics Division, School of Science and Technology, University of Camerino, Via Madonna delle Carceri 9, 62032 Camerino, Italy; emin.mijiti@unicam.it (Y.M.); marco.minicucci@unicam.it (M.M.); 6 Department of Immunology, Genetics and Pathology, Uppsala University, SE-751 85 Uppsala, Sweden; magnus.essand@igp.uu.se

**Keywords:** nanohydrogel, oncolytic virus, cancer immunovirotherapy, drug delivery

## Abstract

Oncolytic viruses (OVs) are emerging as promising and potential anti-cancer therapeutic agents, not only able to kill cancer cells directly by selective intracellular viral replication, but also to promote an immune response against tumor. Unfortunately, the bioavailability under systemic administration of OVs is limited because of undesired inactivation caused by host immune system and neutralizing antibodies in the bloodstream. To address this issue, a novel hyaluronic acid based redox responsive nanohydrogel was developed in this study as delivery system for OVs, with the aim to protect the OVs following systemic administration. The nanohydrogel was formulated by water in oil (W/O) nanoemulsion method and cross-linked by disulfide bonds derived from the thiol groups of synthesized thiolated hyaluronic acid. One DNA OV Ad[I/PPT-E1A] and one RNA OV Rigvir^®^ ECHO-7 were encapsulated into the developed nanohydrogel, respectively, in view of their potential of immunovirotherapy to treat cancers. The nanohydrogels showed particle size of approximately 300–400 nm and negative zeta potential of around −13 mV by dynamic light scattering (DLS). A uniform spherical shape of the nanohydrogel was observed under the scanning electron microscope (SEM) and transmission electron microscope (TEM), especially, the successfully loading of OV into nanohydrogel was revealed by TEM. The crosslinking between the hyaluronic acid chains was confirmed by the appearance of new peak assigned to disulfide bond in Raman spectrum. Furthermore, the redox responsive ability of the nanohydrogel was determined by incubating the nanohydrogel into phosphate buffered saline (PBS) pH 7.4 with 10 μM or 10 mM glutathione at 37 °C which stimulate the normal physiological environment (extracellular) or reductive environment (intracellular or tumoral). The relative turbidity of the sample was real time monitored by DLS which indicated that the nanohydrogel could rapidly degrade within 10 h in the reductive environment due to the cleavage of disulfide bonds, while maintaining the stability in the normal physiological environment after 5 days. Additionally, in vitro cytotoxicity assays demonstrated a good oncolytic activity of OVs-loaded nanohydrogel against the specific cancer cell lines. Overall, the results indicated that the developed nanohydrogel is a delivery system appropriate for viral drugs, due to its hydrophilic and porous nature, and also thanks to its capacity to maintain the stability and activity of encapsulated viruses. Thus, nanohydrogel can be considered as a promising candidate carrier for systemic administration of oncolytic immunovirotherapy.

## 1. Introduction

**Figure 1:**
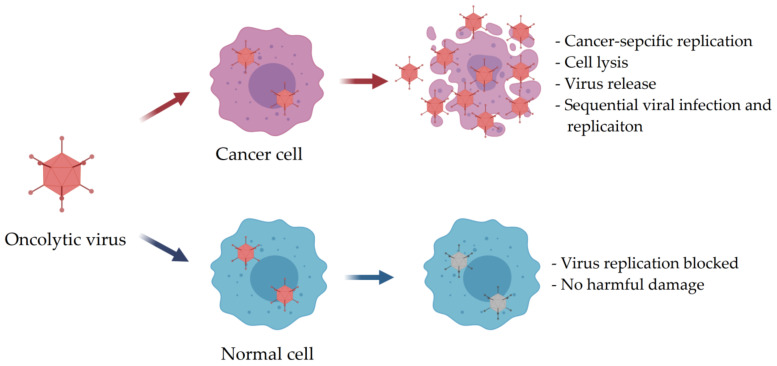
Schematic diagram of cancer cell-specific killing of oncolytic viruses (OVs) (Modified from [13]).

Cancer is one of the most severe diseases and has grown at a remarkable rate globally [1]. Effective strategies for cancer therapy are a pressing need. Over the last few decades, chemotherapy, radiotherapy, and surgery have achieved significant advances as three major treatment options in order to meet the challenge of cancer therapy. In spite of this, the conventional cancer therapies are still limited due to the serious adverse side effects [2,3,4]. Therefore, novel therapeutic approaches characterized by high cancer cell selectivity are urgently needed for cancer therapy. Nowadays, oncolytic immunovirotherapy using oncolytic virus (OV) as a novel and promising anti-tumor therapy has been extensively studied in preclinical and clinical trials [5,6,7,8,9]. Compared with conventional cancer treatments, oncolytic immunovirotherapy provides obvious virtues when administrated OVs directly to tumors. OVs have been intensively investigated as therapeutic agents inducing anti-tumor effect through only infection and competent replication into specific cancer cells without causing unwanted influence on normal cells [10,11,12]. The OVs can be released from previous infected cancer cell once the lysis happened due to the competent replication, then future infect the neighbor cancer cells (Figure 1) [13]. Meanwhile, OVs could also induce systemic anti-tumor immune response [14,15]. In October 2015, the first OV talimogene laherparepvec (T-VEC) was approved by US Food and Drug Administration (FDA) for melanoma immunotherapy [16]. Also, various OVs are being actively developed in different phases of preclinical and clinical trials for different cancers, such as melanoma, glioma, pancreatic, and breast cancers [17]. The most common approach of OVs for cancer immunotherapy is intratumoral administration for solid tumor grown at accessible position for a direct injection, such as head or neck [18,19,20,21]. Nevertheless, for the solid tumor placed at an anatomic location which is inaccessible by a direct injection, or for the metastatic cancers, achieving systemic administration delivery of OVs has attracted an increasing interest. However, the systemic administration of OVs can trigger strongly immune response, inactivate by neutralizing antibodies in the blood stream, subsequently cause decreasing circulation time and finally rapidly eliminated from body, thereby, resulted a low therapeutic efficacy [22,23,24,25,26].

In recent studies, it has been demonstrated that shelter the capsid protein of OVs by polymers or nanoparticles could decrease the anti-viral immune response from host environment, consequently, improve the tumor accumulation of OVs [13]. Several strategies have been studied in order to facilitate the systemic administration of OVs such as conjugation with polyethylene glycol (PEG) [27,28]. Cationic polymers, such as poly (ethylenimine) (PEI) and poly (L-lysine) (PLL) were successfully applied to form nano-complex with OVs via electrostatic interaction [29,30,31,32]. However, the cationic polymers possess high cytotoxicity which could cause side effect to the host [33,34]. It has been evidenced that electrostatic and polar interactions of external ligands in the extracellular environment initiate an avalanche of signal transduction cascade via integrin stimulation in cancer cells, triggering cancer cell proliferation [35] Recently, lipid-based non-viral nanocarrier, liposome and extracellular vehicles (EVs), have attracting attentions to be employed as protective carriers for OVs. The lipid bilayer membrane can provide an aqueous containing cave assisting the encapsulated OVs escape from the immune clearance, but also improving the uptake by target cells [36,37,38]. It is also possible to have un-encapsulated OV remained in the suspension which is still a challenge to purify the OV loaded liposomes/EVs from un-encapsulated OVs [37,39,40,41,42].

Recently, several studies demonstrated that locally injectable hydrogels have the potential for efficient local and long-term delivery of OVs [43,44]. In particular, nanohydrogel, as a nano-dimensional hydrogel, integrates the advantages from both hydrogel systems and nanoparticle systems, thereby, it has been extensively developed and investigated as drug carrier due to their biocompatibility, high water holding capacity, tissue-like mechanical properties, biocompatibility, while also displacing long circulation time in the blood stream, modifiable surface for targeting, and possibility for systemic administration [45]. However, there are no previous studies have used nanohydrogel as systemic delivery system for OVs. Additionally, as a desirable drug delivery system, nanoparticles should have the capacity to protect and preserve the stability of the payload during the delivery in the circulation system, but also trigger the rapid release at the targeted position. Beside temperature, pH or enzyme sensitive system, redox-response drug carriers have demonstrated their promising potential for controlled release, recently [46,47,48,49]. In particular, redox sensitive crosslinking based on disulfide bonds can be cleaved by reduction agent, such as glutathione (GSH) [50]. GSH has been demonstrated that it possesses a relative high concentration level at intracellular or tumor region (2–10 mM) compared with extracellular environment (2–10 μM) [51,52]. Therefore, nanohydrogels containing disulfide bonds has recently attracted increasing attention as intracellular or tumor drug delivery system by taking advantages of this redox potential difference. 

In light of these premises, it will be interesting to further develop a nano-dimensional hydrogel with good biocompatibility, large hydrophilic lumen, proper degradation behavior and easily prone to an appropriate purification process for encapsulation and intravenously delivery of OVs, that was actually the aim of this study. Therefore, a hyaluronic acid based nanohydrogel equipped with redox sensitive disulfide bonds was designed and developed in this study in order to provide a novel system for OVs delivery which can overcome the previously discussed challenges. To our knowledge, using nanohydrogel as delivery system for OVs has never been reported. For these purposes, adenovirus Ad [I/PPT-E1A] (DNA virus) and echovirus Rigvir^®^ ECHO-7 (RNA virus) were selected as two model oncolytic viruses and loaded into the nanohydrogel, respectively. The Ad[I/PPT-E1A], a prostate-specific oncolytic adenovirus, has been engineered to have the E1A gene under the control of a recombinant regulatory sequence designated PPT that comprises a prostate specific antigen (PSA) enhancer, a prostate specific membrane antigen (PSMA) enhancer, and a T-cell receptor γ-chain alternate reading frame protein (TARP) promoter for the specific OV replication in prostate cancer cells, as previously described by Cheng et al. [53]. It has been verified that the Ad[I/PPT-E1A] specifically and efficiently kills prostate cancer cells, in vitro and in vivo [54]. ECHO-7 belongs to the Picornaviridae family, Enterovirus genus, Enteric Cytopathic Human Orphan (ECHO) type 7, group IV, positive-sense single-stranded RNA virus [55,56]. It was selected and adapted for melanoma therapy, and then has been approved and registered in Latvia since 2004. ECHO-7 has been verified to significantly inhibit the development of varies cancers in the clinical trials such as melanoma, gastric cancer, and colorectal cancer, and successfully improve the survival time of the patients [57,58]. These two model OVs were encapsulated to investigate their stability under the formulation conditions, and prove that the nanohydrogel is a suitable delivery system for encapsulation and release of OVs, thereby providing protection for OVs against antibodies in circulation. The escape of OV-loaded nanohydrogels from the host immune system would potentially allow effective intravenous administration of the viral drug that can consequently act against inaccessible cancers as well as metastatic diseases.

## 2. Materials and Methods

### 2.1. Materials

Sodium hyaluronic acid (HA) was purchased from Lifecore Biomedical (Chaska, MN, USA) and used as received. Dithiothreitol (DTT), 1-ethyl-3-(3-dimethylaminopropyl)carbodiimide hydrochloride (EDC•HCl) and L-α-phosphatidylcholine from egg yolk (PC) were bought from Sigma-Aldrich (Schnelldorf, Germany). 3,3′-Dithiobis(propanoic dihydrazide) (DTP) was synthesized according to the procedure described by Vercruysse et al. [59] and characterized by ^1^H-NMR. 3-(4,5-dimethyl-2-thiazolyl)-2,5-diphenyl-2*H*-tetrazolium bromide (MTT) and 3-(4,5-dimethylthiazol-2-yl)-5-(3-carboxymethoxyphenyl)-2-(4-sulfophenyl)-2*H*-tetrazolium (MTS) cell viability assay kits were purchased from Sigma-Aldrich (Milano, Italy) and Promega Corporation (Madison, WI, USA), respectively.

### 2.2. Synthesis of Thiolated Hyaluronic Acid

The synthesis of thiolated hyaluronic acid (HA-SH) was slightly modified according to the procedure described in our previous study [60]. HA was coupled with DTP by carbodiimide chemistry firstly, and then the disulfide bonds were reduced by DTT to obtain free thiol groups as terminal group for HA pending side chains. The number of thiol group substituted per 100 disaccharide units is defined as substitution degree (DS). Briefly, 1 g HA reacted with 245.54 mg DTP in 100 mL ultrapure water at room temperature and pH 4.75. EDC•HCl (197.48 mg) was added as a carboxyl activating agent. The reaction was sustained for 48 h and then stopped by increasing the pH to 7. Subsequently, 4.07 g DTT was added to the reaction mixture as reducing agent and the pH was changed to 8.5. The reaction continued for 24 h and final completed by decreasing the pH to 3.5. The reaction solution was purified by dialysis (Mw cutoff = 12–24 kDa) against 100 mM sodium chloride (NaCl) solution at 4 °C, pH 3.5 for 3 days, then dialyzed against deionized water for additional 24 h. The final product was isolated as dry powder by lyophilization (Freeze dryer, FreeZone, Labconco, Kansas City, MO, USA) and stored at −20 °C. 30% DS of thiol groups was obtained and characterized by ^1^H-NMR in D_2_O, δ in ppm: 2.03 (3H, -NHC(=O)C*H_3_*); 2.67(2H, -C*H_2_*CH_2_SH); 2.85 (2H, -CH_2_C*H_2_*SH); 3.34–4.5 (protons of hyaluronic acid main chains). 

### 2.3. Proton Nuclear Magnetic Resonance (^1^H-NMR)

The chemical structure and substitution degree (DS) of thiol group of HA were characterized by proton nuclear magnetic resonance (^1^H-NMR, Varian Mercury plus 400, Crawley, UK) using deuterium oxide (D_2_O) as solvents. Chemical shifts was referred to the solvent peak δ = 4.79 ppm for D_2_O.

### 2.4. Oncolytic Viruses

The genetically modified oncolytic adenovirus Ad[I/PPT-E1A] was kindly provided by Prof. Magnus Essand (Uppsala, Sweden) in stocks of 1 × 10^12^ particle forming units (PFU) in PBS. All vials were stored at −80 °C and freshly thawed on ice before each experiment. 

Rigvir^®^ ECHO-7 was provided by the marketing authorization holder SIA Latima (Riga, Latavia). It is a PBS solution of an adapted and selected ECHO-7 virus stain at a titer ≥ 1 × 10^6^ TCID50/mL stored at −80 °C and transported frozen. 

### 2.5. Formulation of Empty/OV-Loaded Nanohydrogel 

**Figure 2:**
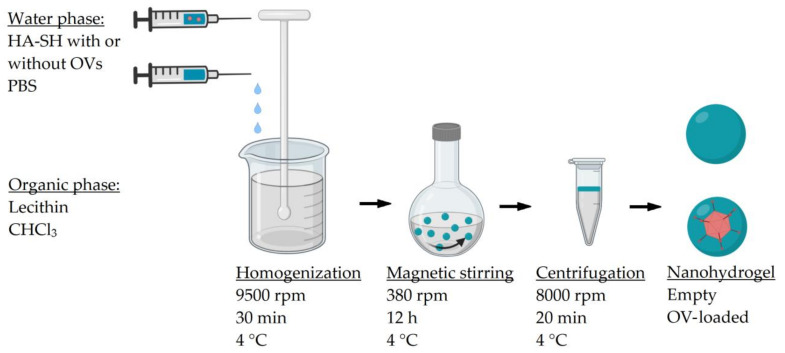
Schematic diagram of empty nanohydrogel and OV-loaded nanohydrogel formulation by water in oil (W/O) nanoemulsion optimized method.

Empty nanohydrogel and OVs-loaded nanohydrogel were prepared by W/O nanoemulsion method (Figure 2). In details, the viruses were firstly suspended in a PBS solution at a concentration of 6.8 × 10^9^ PFU/mL and 7.9 × 10^5^ PFU/mL for Ad[I/PPT-E1A] and ECHO-7, respectively. Then, HA-SH was dissolved in pure PBS or virus PBS suspension at a concentration of 5% *w/v* to act as water phase for the preparation of empty or OV-loaded nanohydrogel, separately. Meanwhile, lecithin was dissolved in chloroform (CHCl_3_) at a concentration of 2.5% *w/v* as organic phase. Subsequently, water phase was added dropwise into organic phase along homogenization (Ultra-Turrax^®^ T25 digital, IKA, Staufen, Germany) at a speed of 9500 rpm for 30 min at 4 ± 1 °C. The formed nanoemulsion was kept at 4 ± 1 °C overnight under gentle stirring at 380 rpm to allow the nanohydrogel crosslinking. The next day, the nanohydrogel was collected by centrifugation (High speed micro-centrifuge, D3024R, Scilogex, Rocky Hill, CT, USA) at 8000 rpm for 20 min. The upper water phase containing nanohydrogels was collected and washed by CHCl_3_ to remove the residual surfactant. Subsequently, 7% sucrose PBS solution was added and residual CHCl_3_ was removed by stirring under the fume hood for 2 h. Finally, the empty nanohydrogel or OV-loaded nanohydrogel was stored at −80 °C with 7% sucrose as cryoprotectant.

### 2.6. Dynamic Light Scattering (DLS)

The particle size, polydispersity index (PDI) and zeta potential were characterized by dynamic light scattering (DLS,) using a Zetasizer Nano-S90, Malven instruments (Malvern Panalystical, Malvern, UK) at a fixed 90° scattering angle at 25 °C. The measurements were performed in triplicate.

### 2.7. Scanning Electron Microscope (SEM)

The morphology and particle size of the nanohydrogel were studied by a scanning electron microscope (field emission-SEM Zeiss Σigma 300, Zeiss, Oberkocken, Germany). SEM sample stage was prepared by placing a double-sided adhesive carbon tape on an aluminum stub. A small amount of nanohydrogel lyophilized powder was placed on the sample stage. Subsequently, the sample was sputtered under vacuum with a chromium layer of approximately 100 Å thickness (Quorum Q150T ES, Quorum Technologies, Lewes, UK).

### 2.8. Transmission Electron Microscope (TEM)

A transmission electron microscope (TEM, Tecnai G2 F30 S-TWIN, Thermo Scientific, Waltham, MA, USA) at an acceleration voltage of 300 keV was used for observing the interior structure of empty and OV-loaded nanohydrogels. A copper grid was covered with a layer of carbon and treated by hydrophilic electric ionic. Then, a droplet of nanohydrogel suspension was placed on the treated copper grid and stained by 0.75% phophatungstic acid. Excess water of sample was removed by filter paper carefully dried at room temperature.

### 2.9. Raman Spectroscopy

Cross-linked nanohydrogel and HA-SH polymer were characterized by Raman spectroscopy. Raman experiments were performed using a micro-Raman spectrometer (iHR320, Horiba, Kyoto, Japan) in backscattering geometry and a microscope (Olympus BXFM-ILHS, Olympus Corporation, Tokyo, Japan). 

A diode-pumped solid state laser of 532 nm emission wavelength was used as the excitation source. Raman scattering light was collected using a 50× microscopy objective and dispersed with 600 grooves mm^−1^ grating and detected using a cooled charge coupled device array detector (Horiba Syncerity, Horiba, Japan). 

### 2.10. Redox Sensitive and Stability Test of the Nanohydrogel

Glutathione (GSH) was used as reduced agent to detect redox responsive ability of nanohydrogel. Nanohydrogels were incubated at 37 °C in pH 7.4 PBS in the presence of 10 μM and 10 mM GSH as mimicking the extracellular environment and reducing intracellular environment, respectively, while in pH 7.4 PBS without GSH as negative control. The redox responsiveness was evaluated by determining relative turbidity of these two groups nanohydrogel by DLS along with time. The stability of the nanohydrogel in normal physiological environment was examined by monitoring the particle size and PDI in pH 7.4 PBS at 37 °C along with time in order to verify the systemic stability of the nanohydrogel after administration. 

### 2.11. Cell Culture

HT-29 (ATCC^®^ HTB-38^™^, American Type Culture Collection, Manassas, VA, USA) colorectal carcinoma cell line [61] was selected for the in vitro cytotoxicity study of ECHO-7 virus. The HT-29 colorectal carcinoma cell line was grown in Dulbecco’s modified eagle medium (DMEM) supplemented with 10% fetal calf serum (FCS), 1% penicillin-streptomycin (PS) and 2 mM glutamine cultured at 37 °C and 5% CO_2_ incubator. 

LNCaP metastatic prostate cancer cell line was provided by Professor Magnus Essand [62] and selected for the in vitro cytotoxicity study of adenovirus Ad[I/PPT-E1A]. The LNCaP metastatic prostate cancer cell line was grown in Roswell Park Memorial Institute (RPMI) 1640 supplemented with 10% fetal calf serum (FCS) and 1% penicillin-streptomycin (PS) cultured at 37 °C and 5% CO_2_ incubator. Cells were routinely tested for mycoplasma and authenticated using PCR.

### 2.12. In Vitro Cytotoxicity Assay

The in vitro cytotoxicity of empty nanohydrogel, Ad[I/PPT-E1A] virus and Ad[I/PPT-E1A]-loaded nanohydrogel against LNCaP cells was evaluated by MTT assay. LNCaP cells were cultured in 12-well plate at a density of 5 × 10^4^ cells per well in a 5% CO_2_ incubator at 37 °C overnight. The next day, the old medium was replaced with RPMI containing different concentration of pure Ad[I/PPT-E1A] virus, empty nanohydrogel or Ad[I/PPT-E1A]-loaded nanohydrogel, respectively, and co-incubated in 5% CO_2_ incubator at 37 °C. After 3 or 5 incubation days, the medium was removed and the cells were three times washed by PBS. Then, 375 μL MTT PBS solution (2.5 mg/mL) was pipetted into each well and further incubated in 5% CO_2_ at 37 °C for 4 h. The resulting formazan was solubilized by DMSO for 5 min and read by microplate reader at 540 nm (SpectraMax iD3, Molecular Devices, San Jose, CA, USA).

The viability of HT 29 colorectal carcinoma cells was inspected by MTS assay to characterize the cytotoxicity of ECHO-7 virus, empty nanohydrogel, and ECHO-7-loaded nanohydrogel. HT-29 cells were cultured in 96-well plate at a density of 1 × 10^4^ cells per well in a 5% CO_2_ incubator at 37 °C overnight. The next day, the old medium was replaced with DMEM containing different concentration of ECHO-7 virus, nanohydrogel suspension with or without ECHO-7 virus loaded, respectively, and co-incubated in 5% CO_2_ at 37 °C. After 5 or 7 incubation days, the medium was removed and the cells were three times washed by PBS. Twenty μL MTS solution was pipetted into each well and future incubated in 5% CO_2_ at 37 °C for 1 h. Subsequently, the plate was read by microplate reader at 490 nm (SpectraMax iD3, Molecular Devices, San Jose, CA, USA).

The cell viability percentage of the MTT and MTS assay was calculated according to Equation (1).

(1) $$\mathrm{Cell}\;\mathrm{viability}\%=\frac{Average\;absorbance\;of\;triplicate\;treated\;wells}{Average\;absorbance\;of\;triplicate\;untreaated\;wells}\times 100\%$$

### 2.13. Statistical Analysis

The experimental results were reported as mean ± standard deviation. One-way ANOVA was applied for statistical analysis via SPSS software (24.0 version, IBM, Chicago, IL, USA), while a *p* value less than 0.05 was considered statistically significant. 

## 3. Results and Discussion

### 3.1. Encapsulation of Oncolytic Adenovirus into the Nanohydrogel

The nanohydrogel was developed by water in oil (W/O) nanoemulsion method using synthesized HA-SH polymer (Figure 2) and characterized from the physicochemical point of view. HA-SH polymer was dissolved into OVs PBS suspension and then added dropwise to the chloroform along with homogenization at 9500 rpm for 30 min to form nanoemulsion. The homogenization time and speed was screened from different conditions in order to process the nanohydrogel with a suitable size which is bigger than naked OV for protection, but also smaller than 400 nm in order to penetrate the tumor vascular system [63,64]. Then, the nanoemulsion was gently stirred overnight for the formulation of disulfide cross-linking among thiol groups of HA-SH polymer. During the cross-linking procedure, the OVs are supposed to be maximum entrapped into the nano water drop due to the viscosity of HA polymer, but also the hydrophilicity nature of OVs, therefore avoiding the harsh and degrading environment of the outer organic phase. In time, OVs are expected to be stabilized inside the nanohydrogel by simultaneous disulfide crosslinking of the nanohydrogel. Chloroform was chosen as an organic phase in order to form a nanoemulsion benefiting the incompatibility with water. Additionally, it can inactivate the un-encapsulated OVs via denaturing the lipid or protein capsid. Thereby, the OV-loaded nanohydrogel can be easily separated from un-encapsulated OVs by centrifugation to break the nanoemulsion. In order to maximally retain the activity of OVs, the homogenization and cross-linking procedure were carried out at 4 °C. Additionally, a control group was operated with same homogenization parameters but did overnight cross-linking at room temperature. As expected, there was no oncolytic activity of OV-loaded nanohydrogel detected via in vitro cytotoxicity assay which indicated that operating temperature is one of the most important conditions for OV encapsulation. 

The particle size, PDI and zeta potential of the nanohydrogels were characterized by DLS and summarized in Table 1. The results demonstrated that all the nanohydrogels possessed an approximate particle size of 300–400 nm, with a relatively uniform polydispersity distribution as supported by the low PDI. In details, the particle sizes of the OV loaded nanohydrogels were 362 ± 19 nm and 347 ± 10 nm for Ad[I/PPT-E1A] and ECHO-7-loaded nanohydrogels, respectively; they were both around 50 nm smaller in diameter compared with respective empty nanohydrogels (426 ± 12 nm and 431 ± 13 nm). The greater size of the OV encapsulated nanohydrogels with the respect to the empty ones can be easily explained as follows: The empty nanohydrogel is composed of HA-SH polymer able to undergo to a swelling of large extent once in the hydrated state. OVs are composed of a nucleoprotein core and an icosahedral nonenveloped capsid possessing a size around 50–100 nm [65,66], which do not make a distinct variation of volume in the aqueous environment. The interior cross-linked matrix of the OV-loaded nanohydrogel was partly occupied by OVs, as a result, represented less swelling in the hydrated state. The replacement of the HA-SH swelling polymer of the nanohydrogel with the virus reduces the swelling tendency of the nanohydrogel, thus leading to a particle smaller in size.

**Figure 3:**
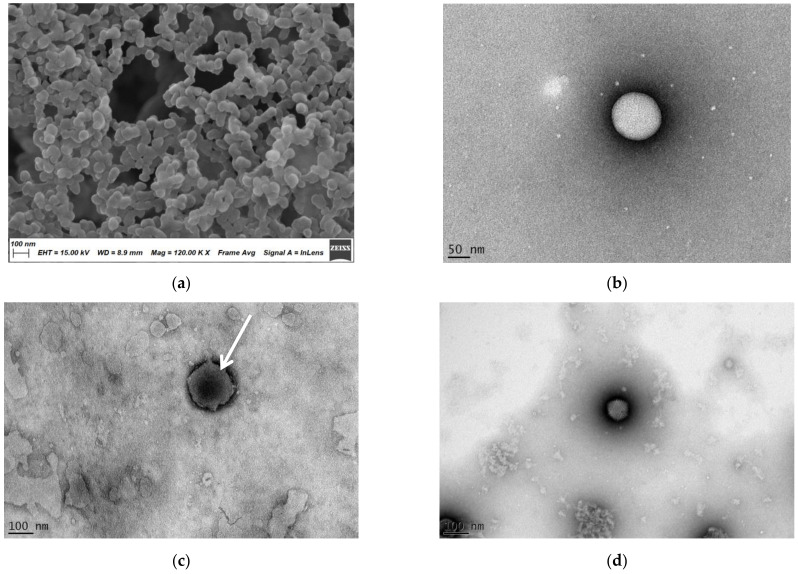
Scanning electron microscopy (SEM) and transmission electron microscopy (TEM) characterization: (**a**) SEM photo of the empty nanohydrogel; (**b**) TEM photo of the empty nanohydrogel; (**c**) TEM photo of Ad[I/PPT-E1A]-loaded nanohydrogel; (**d**) TEM photo of naked Ad[I/PPT-E1A] virus.

To investigate the external and internal morphology of the nanohydrogels, the nanohydrogels were further observed by SEM and TEM (Figure 3). As shown in Figure 3a, the nanohydrogels adopted a spherical shape with a smooth surface under the SEM. The nanohydrogel presented a uniform particle size distribution of around 100 nm, which is much smaller than the size measured by DLS, but in a good agreement with the one measured by TEM (Figure 3b). The explanation of the discrepancy in the nanohydrogel particle size is due to the differences in the experimental conditions: During DLS measurement, nanohydrogel swells in the aqueous environment, while, on the contrary, SEM and TEM are performed in anhydrous conditions and thus the nanohydrogel incurs in an extreme shrunk that impacts the size reduction. 

Interestingly, the successfully encapsulation of Ad[I/PPT-E1A] virus was distinctly observed under the TEM (Figure 3c). While pure Ad[I/PPT-E1A] virus (Figure 3d) and empty nanohydrogel (Figure 3b) images were used as positive and negative control. According to the TEM image of pure Ad[I/PPT-E1A], the icosahedral capsid was clearly observed with a size around 80 nm, consistent with previous study [67]. Compared with the homogeneous transparency core of empty nanohydrogel (Figure 3b), there is an apparent shadow where the arrow points revealed in the center of Ad[I/PPT-E1A]-loaded nanohydrogel (Figure 3c) which suggests effective OVs encapsulation. It also turns out that the virus was encapsulated into the nanohydrogel one to one. Additionally, in Figure 3c, the shape of the virus icosahedral capsid was exposed along with the shrinkage of the nanohydrogel but with a bigger size compared with naked OV (Figure 3d) which further indicated the successful encapsulation of Ad[I/PPT-E1A] virus.

**Figure 4:**
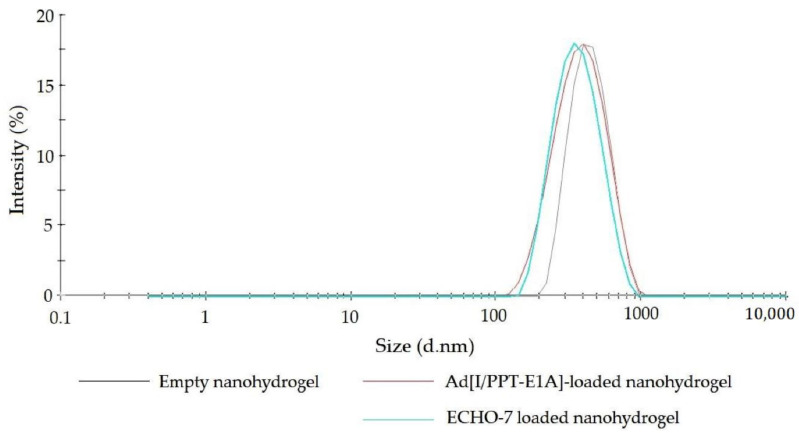
Size distribution curve of developed nanohydrogels examined by DLS.

Concerning the nanoparticle charge, Table 1 clearly showed that there was no difference in zeta-potential between empty nanohydrogel and OV loaded nanohydrogel, as expected. Zeta potential is the surface charge of nanoparticles which is an important parameter to evaluate for the colloidal stability of the nanoparticle system [68,69]. The formulated nanohydrogels displayed negative zeta potential (~−13 mV) which depends on the contribution of by both hyaluronic acid and the residual surfactant lecithin. It is of paramount importance for the nanohydrogel administration and shelf life to evaluate the zeta potential in order to avoid aggregation and preserve stability in suspension. It has been demonstrated that a nanoparticle system possessing a zeta potential approximate ±30 mV can lead to a good stabilization of the suspension, and smaller than 5 mV may cause severe undesired aggregation [70,71,72]. Nonetheless, the absence of aggregation of our nanohydrogel in water was proven by DLS immediately after preparation, that shows only single peak was detected without particle populations larger in size range confirming the absence of aggregates (Figure 4). The stability of the nanohydrogel suspension was evaluated by particle size and PDI monitoring for 5 days and the results are described in following (Section 3.3). As a consequence, these preliminary results lead to envisage a good colloidal stability of nanohydrogel system in the circulatory system after administration.

### 3.2. The Nanohydrogel Is Successfully Cross-Linked by Disulfide Bonds

**Figure 5:**
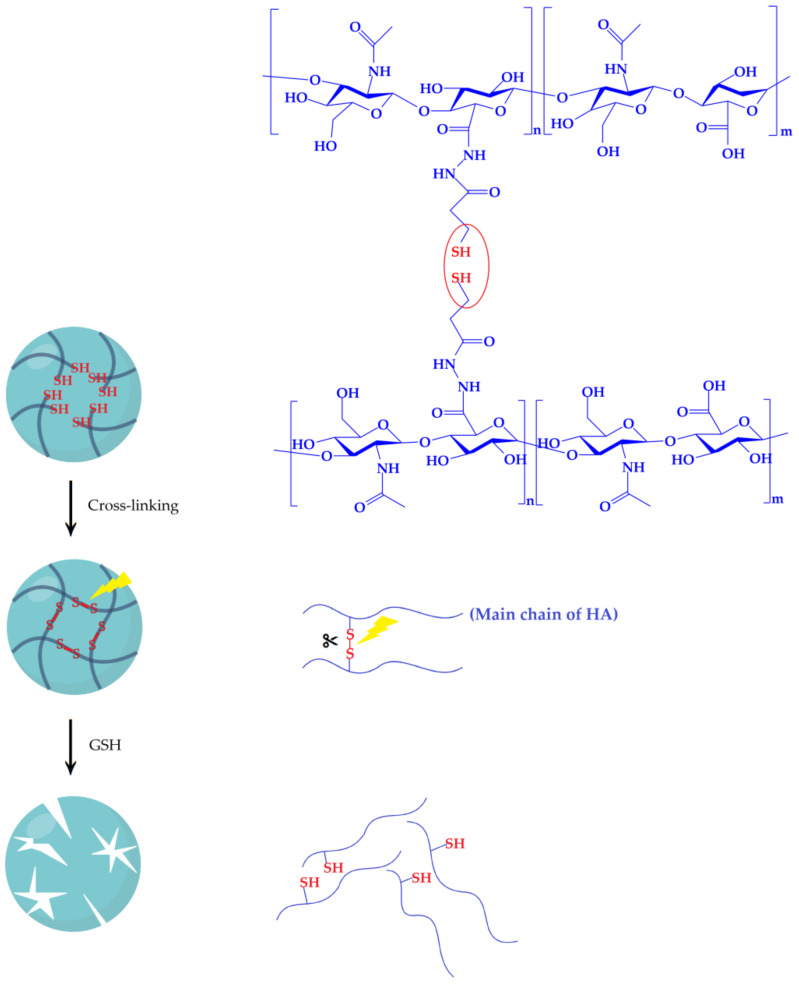
Illustration of cross-linking and redox response degradation behavior of the nanohydrogel.

**Figure 6:**
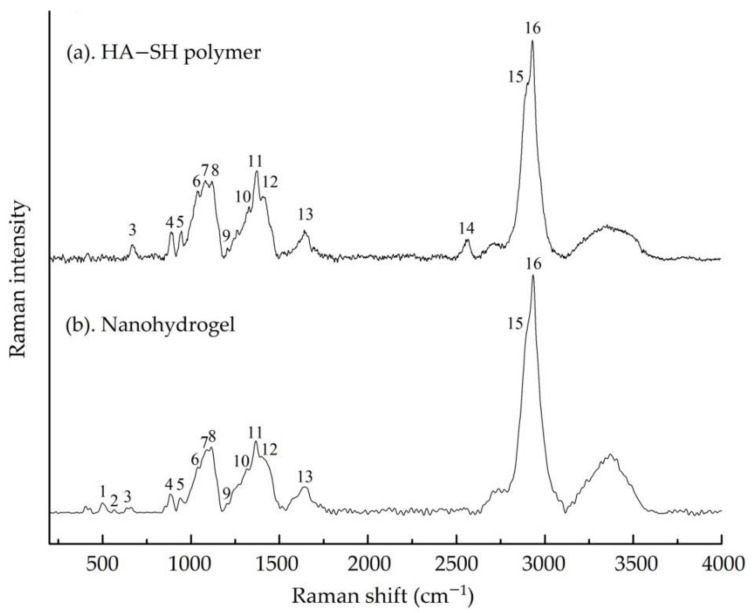
Raman spectra of (**a**) thiolated hyaluronic acid (HA-SH) polymer and (**b**) nanohydrogel.

As illustrated in the Figure 5, the formulation of the nanohydrogel was engineered getting the contribution of the disulfide bonds derived from the HA-SH polymer. Raman spectroscopy was conducted to verify disulfide cross-linking of the nanohydrogel. HA-SH (DS 30%) polymer was examined as a reference Raman spectrum for the nanohydrogel. Table 2 summarized the Raman bands observed in HA-SH polymers and nanohydrogel, and also the corresponding data cited from literatures. As shown in Figure 6a, Raman spectrum of HA-SH polymer exhibiting all the characteristic peaks of hyaluronic acid [73,74,75] with a specific peak at 2557 cm^−1^ which is assigned to thiol group [76,77].The Raman spectrum of the nanohydrogel (Figure 6b) maintains the characteristic peaks of hyaluronic acid, which proves that hyaluronic acid represents almost the majority of the nanohydrogel. Indeed, the comparison between spectra of the HA-SH polymer and the nanohydrogel reveals the disappearance of the thiol group peak, and the appearance of two new peaks at 501 and 565 cm^−1^ ascribed to the stretching vibration and bending vibration of the new formed disulfide bonds. This result indicates that all the thiol groups of HA-SH polymer were consumed and the nanohydrogels were chemical cross-linked by disulfide bonds derived from the thiol groups of HA-SH polymer.

### 3.3. The Nanohydrogel Possesses Stability and Redox Responsiveness

The nanohydrogel was cross-linked by disulfide bonds which has been identified can be cleaved by reduce agent, thereby inducing degradation of nanohydrogel in the environment with high GSH concentration [79]. To study the redox response ability of the nanohydrogel, different amount of GSH were added into the nanohydrogel PBS suspension pH 7.4, to achieve the final GSH concentrations of 10 μM and 10 mM in order to mimic the extracellular and intracellular circumstances, respectively [80]. The turbidity of the samples was monitored by detecting the scattering intensity using DLS. The degradation of nanoparticles can be directly detected according to reduce of sample turbidity and a relative turbidity % is calculated according to the Equation (2) in order to study the dynamic degradation progress [81].

(2) $$\mathrm{Relative}\;\mathrm{turbidity}\;\%=\frac{Real\;time\;scattering\;intensity\;}{Orignal\;scattering\;intensity}\times 100\%$$

**Figure 7:**
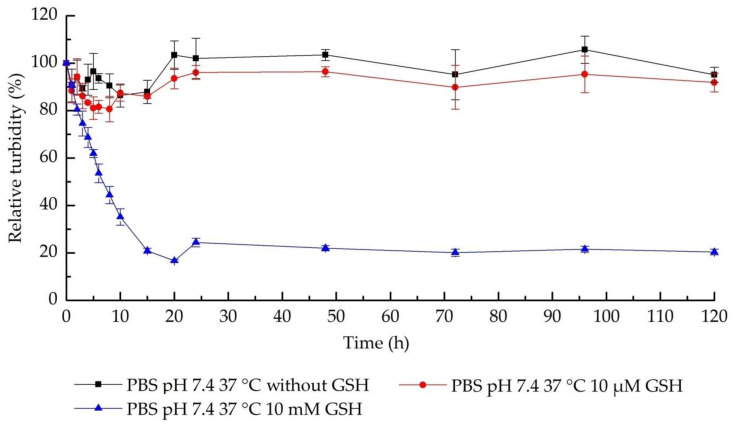
Characterization of redox response of nanohydrogel by relative turbidity measurements.

**Figure 8:**
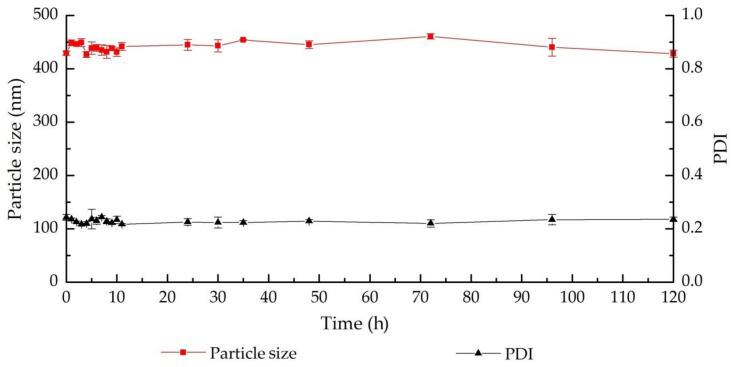
Characterization of the nanohydrogel stability in phosphate buffered saline (PBS, 10 mM, pH 7.4) at 37 °C by particle size and PDI determination.

As displayed in Figure 7, almost no obvious change in the relative turbidity was determined in the nanohydrogel suspension with 10 μM GSH in 20 h compared with the negative control (absence of GSH), demonstrating that the nanohydrogel is stable in the extracellular environment, i.e., blood circulation. Then, once the concentration of GSH increased to 10 mM, the relative turbidity of the sample showed a rapid drop in the first 5 h and then decreased gradually in next several hours, which suggests that the particle size and/or the particle number were reducing due to the reductive cleavage of the disulfide bonds in the high concentration of GSH. The nanohydrogel is supposed to finally degraded into single HA chain that can be efficiently cleared from body circulation due to its hydrophilicity and biocompatibility (Figure 5) [82]. Additionally, as demonstrated in Figure 8, the developed nanohydrogel provide a stable particle size and PDI around 450 nm and 0.25 in the time range considered for the study, which further confirmed that the nanohydrogel possessed a good stability in the normal physiological environment. The results indicate that this nanohydrogel system can capably protect the payload from leaking out during the delivery, while rapidly releasing the payload in the reductive environment, such as tumor cells [83]. Therefore, it can be concluded that this nanohydrogel possesses significant redox responsiveness and promising potential to be developed for intracellular therapy or cancer treatment.

### 3.4. Oncolytic Activity of OV-Loaded Nanohydrogels

The cytolytic ability of OV-loaded nanohydrogel was determined by measuring the cytotoxicity in the corresponding cancer cell lines according to the specificity of the encapsulated OVs. As described in the introduction (Figure 1), the anti-cancer mechanism of OVs is to replicate following cellular infection of cancer cells and ultimately resulting in oncolysis. The oncolysis effect indicates that OV can efficiently replicate in the specific cell lines and then be cytolytic. A lag time must be generally considered prior to observe the cythopathic effect on cancer cells after the OVs treatment, and this is explained with the necessity to complete a sufficient cytolytic replication of OVs [13,84].

**Figure 9:**
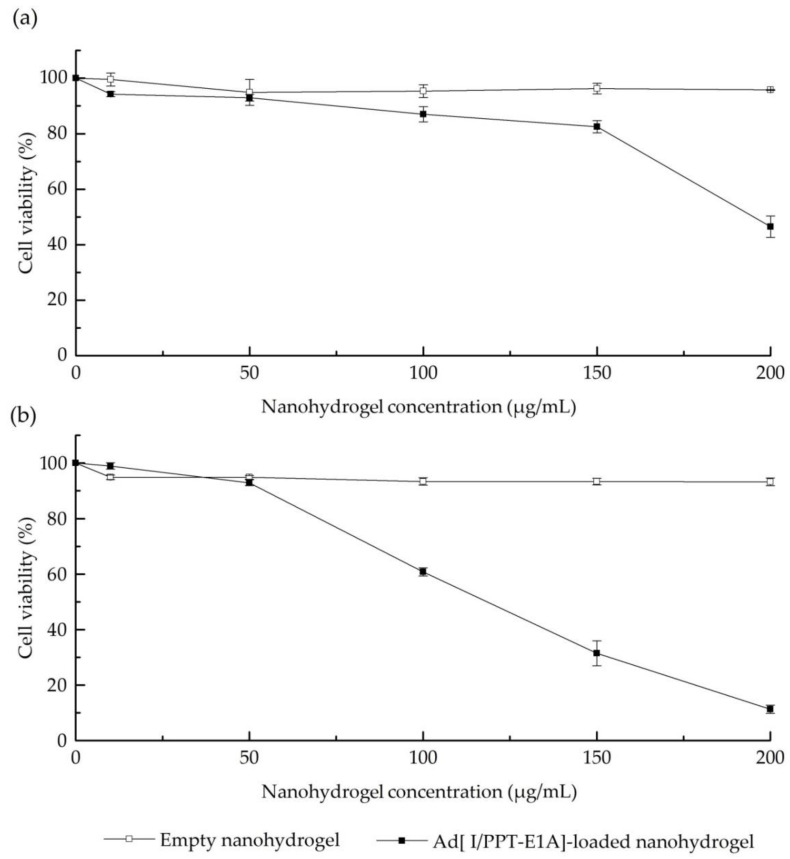
3-(4,5-dimethyl-2-thiazolyl)-2,5-diphenyl-2*H*-tetrazolium bromide (MTT) cytotoxicity assays in LNCaP metastatic prostate cancer cell line of: (**a**) Empty nanohydrogel vs. Ad[I/PPT-E1A]-loaded nanohydrogel after 3 days infection; (**b**) Empty nanohydrogel vs. Ad[I/PPT-E1A]-loaded nanohydrogel after 5 days infection.

The in vitro MTT cytotoxicity assay of Ad[I/PPT-E1A]-loaded nanohydrogel was carried out against LNCaP metastatic prostate cancer cell line due to the prostatic cancer specificity of Ad[I/PPT-E1A] [54]. Cytolytic effect was observed in the LNCaP cells treated with Ad[I/PPT-E1A]-loaded nanohydrogel, at concentration of at least 100 μg/mL after 3 days infection (Figure 9a). This result is in agreement to the literature that showed that the pure Ad[I/PPT-E1A] virus possesses evident toxicity in LNCaP cell line after 3 days transduction [53]. Cell viability markedly decreased from 3 to 7 days once concentration rose to 100, 150, and 200 μg/mL (Figure 9b). The Ad[I/PPT-E1A]-loaded nanohydrogel showed the most efficient cythopathic effect after 7 days at a concentration of 200 μg/mL. In contrast, the empty nanohydrogel did not cause cytotoxicity at the same concentration range of the Ad[I/PPT-E1A]-nanohydrogel, which confirmed that the cells death was actually induced by the encapsulated OVs. This result proves that, despite the encapsulation procedure involves the use of chloroform, the encapsulation procedure did not compromise the cytolytic ability of Ad[I/PPT-E1A] and confirms its ability to successful transduce the LNCaP prostatic cancer cells, causing efficient oncolysis. In addition, even if we hypothesized that most of the Ad[I/PPT-E1A] viruses have been encapsulated into the nanohydrogel, in our experiments, we did not exclude that, some viruses escaped from the HA water core during the nanohydrogel formulation and thus could be the responsible for cytolytic activity. To further confirm that the cytolytic effect was caused by encapsulated Ad[I/PPT-E1A] but not free ones, the activity of chloroform treated Ad[I/PPT-E1A] was evaluated by MTT assay against LNCaP cancer cells. Even if literature reports that only around 20% cell viability was observed in LNCaP prostate cancer cells treated by Ad[I/PPT-E1A] at an MOI of 1 after 5 days [53], in our study no cytotoxicity was observed by chloroform treated Ad[I/PPT-E1A] at the MOI from 1 to 25 after 5 days. It verified that the assumed free viruses escaped from water core were inactivated once exposed overnight in the chloroform that could denature the lipid or protein capsid of OVs. This results indicated that this W/O nanoemulsion method can efficiently encapsulated Ad[I/PPT-E1A] virus into the nanohydrogel and simultaneously isolated from free viruses. Furthermore, according to the GSH results reported in the previous paragraphs, this nanohydrogel shows stability in the mimic extracellular environment, while degradation occurs in the reductive conditions such as intracellular or tumor environment, due to the redox responsive property. Since it has been proved that redox-responsive nanosystem can control the release of payload into the cancer cells after internalization [79,85,86], thereby, we supposed that the resulting cytotoxicity of Ad[I/PPT-E1A] could only possible after the Ad[I/PPT-E1A] nanohydrogels up taken and then cytoplasmic Ad[I/PPT-E1A] release. Our study proves that the Ad[I/PPT-E1A] was successfully encapsulated into the nanohydrogel, then up taken into the cancer cells and finally released by nanohydrogel degraded intracellularly, with consequent cancer cells apoptosis.

**Figure 10:**
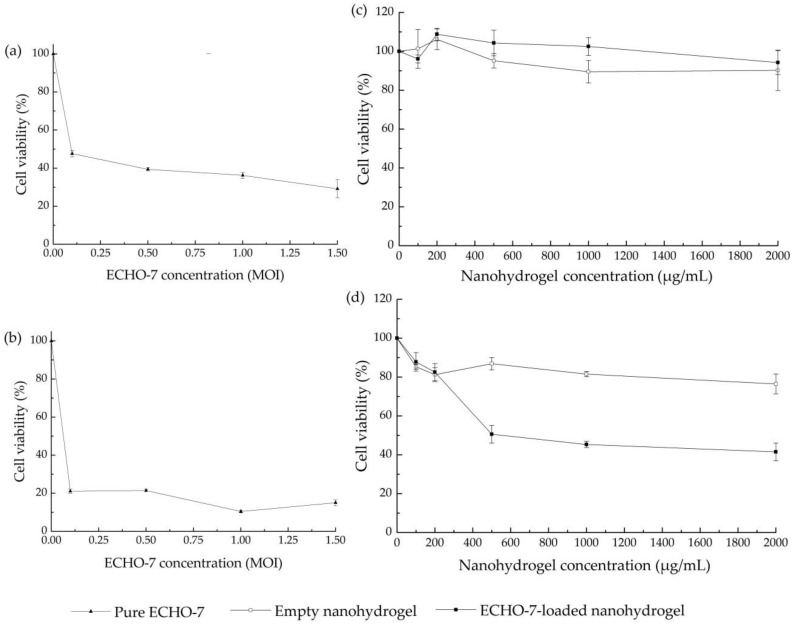
3-(4,5-dimethylthiazol-2-yl)-5-(3-carboxymethoxyphenyl)-2-(4-sulfophenyl)-2*H*-tetrazolium (MTS) cytotoxicity assays in HT29 colon cancer cell line of: (**a**) ECHO-7 after 5 days infection; (**b**). ECHO-7 after 7 days infection; (**c**) Empty nanohydrogel vs. ECHO-7-loaded nanohydrogel after 5 days infection; (**d**) Empty nanohydrogel vs. ECHO-7-loaded nanohydrogel after 7 days infection.

To further verify the ability of nanohydrogel for OV encapsulation, in vitro MTS cytotoxicity assay was carried out for ECHO-7 encapsulated nanohydrogels against HT29 colon cells. The ECHO-7 virus is an OV that has been approved and registered in Latvia since 2004 for cutaneous melanoma [56]. According to the recent case-report, it improved colorectal cancer treatment in combination with other drugs [57]. However, limited data about in vitro cytotoxicity of ECHO-7 against HT 29 colon cells are available in existing researches, the cytolytic ability of pure ECHO-7 viruses were studied first. Our results indicate that pure ECHO-7 virus induced an evident cytotoxic effect after 5 days of treatments at a different range of MOIs from 0.25 to 1.5, and then caused the most efficient cythopathic effect after 7 days of virus transduction (Figure 10a,b). In contrast, there was no evident cytotoxicity observed on HT 29 colon cells after 5 days co-incubation for all tested concentrations of ECHO-7-loaded nanohydrogels (Figure 10c). Cell viability was reduced of around 50% by the treatment with 500, 1000, and 2000 μg/mL EHCO-7-loaded nanohydrogel once the transduction phase was prolonged to 7 days (Figure 10d). Compared with pure ECHO-7 virus replicating in 5 days, the efficacy of anti-cancer activity was delayed approximately of two days. This observation can be possibly explained by the fact that the internalization procedure and redox triggered degradation delayed the virus replication, assembly and release [37,42]. Our study also proves again the successful encapsulation and controlled release of OVs by this nanohydrogel delivery system.

In conclusion, these results indicate that different OVs are stable at the W/O nanoemulsion conditions used for the nanohydrogel formulation as proven by their cytolytic ability, and that they can be successfully encapsulated into the nanohydrogel. Results also suggest that the OV-loaded nanohydrogel can efficiently controlled release of the OV into cancer cells, able to infect them, and induce cell lysis in specific cancer cell lines.

## 4. Conclusions

In this work, a hyaluronic acid based redox-responsive nanohydrogel was developed as delivery system for OVs. One DNA OV Ad[I/PPT-E1A] and one RNA OV ECHO-7 were used as model viral drugs. The results indicated that these two different OVs were both successfully encapsulated into the nanohydrogel, redox-stimulate released into cells and finally killed the specific cancer cells. This study investigates that, besides liposome and EVs, nanohydrogel can also be considered as a novel candidate to protect and carry OVs to their target location, such as tumors, without being neutralized by the immune system. Additionally, this W/O nanoemulsion method can provide insights in the preparation of nanoparticle system for OVs. It takes advantages of water phase to protect OVs during the cross-linking and inactive the un-encapsulated OVs via organic solvent, thereby improve the purification procedure. These proof-of-principle results suggest that this nanohydrogel delivery system has potential to be used as OV protector and carrier in order to achieve systemic administration and site-specific targeting of oncolytic immunovirotherapy. Further studies should be developed to determine the oncolytic activity and also the anti-virus neutralization ability of the OV-loaded nanohydrogel, in vivo. Furthermore, adding cancer targeting moieties or peptides/proteins on the surface of the nanohydrogel is also significant to further develop this nanohydrogel into site-specific intracellular delivery system.

## Figures and Tables

**Table 1:** Particle size, polydispersity index (PDI) and zeta potential of empty and OV-loaded nanohydrogels characterized by dynamic light scattering (DLS).

Name	Particle Size (nm)	PDI	Zeta Potential (mV)
Empty nanohydrogel	426 ± 12	0.29 ± 0.03	−13.2 ± 1.6
Ad[I/PPT-E1A]-loaded nanohydrogel	362 ± 19	0.29 ± 0.03	−12.7 ± 0.9
Empty nanohydrogel	431 ± 13	0.24 ± 0.01	−13.2 ± 3.2
ECHO-7-loaded nanohydrogel	347 ± 10	0.26 ± 0.01	−13.1 ± 2.9

**Table 2:** Assignment of observed Raman bonds in the HA-SH polymer and the nanohydrogel.

Raman Shifts (cm^−1^)				Assignment	
Measurements			References	Bonds	Contributed Polymer
No.	HA-SH	Nanohydrogel			
1	--	501	498 [76,77,78]	S-S _str_	disulfide cross-linked core
2	--	565	560 [76,77,78]	S-S _bend_	disulfide cross-linked core
3	666	662	660 [76]	C-S _str_	linkage of thiol group on HA chain
4	889	883	889 [73,74,75]	--	HA
5	941	940	949 [73,74,75]	--	HA
6	1037	1038	1047 [73,74,75]	C-C_str_ C-O_str_	HA
7	1084	1090	1091[73,74,75]	C-OH_bend_ acetyl group	HA
8	1120	1118	1125 [73,74,75]	C_(4)_-OH_bend_ C_(4)_-H_bend_	HA
9	1207	1206	1205 [73,74,75]	CH_2twist_	HA
10	1329	1316	1328 [73,74,75]	C-H_bend_ Amide III	HA
11	1374	1366	1372 [73,74,75]	C-H_bend_	HA
12	1409	1405	1406 [73,74,75]	C-N_str_ C-H_def_	HA
13	1645	1644	1660 [73,74,75]	C=C Amide I	HA
14	2557	--	2574 [76]	-SH_str_	thiol group
15	2905	2905	2904 [73,74,75]	C-H_str_	HA
16	2933	2933	2933 [73,74,75]	N-H_str_	HA

## Data Availability

Not Applicable.
